# Importance of Different Parameters for Monitoring Dogs with *Leishmania infantum* Infections in a Non-Endemic Country

**DOI:** 10.3390/pathogens14121282

**Published:** 2025-12-12

**Authors:** Melanie Kaempfle, Roswitha Dorsch, Yury Zablotski, Katrin Hartmann, Michèle Bergmann

**Affiliations:** LMU Small Animal Clinic, Centre for Clinical Veterinary Medicine, Ludwig Maximilian University of Munich (LMU Munich), 80539 Munich, Germany

**Keywords:** canine leishmaniosis, CanL, monitoring, relapse, antileishmanial treatment, *Leishmania* antibodies, allopurinol, lymphadenopathy, proteinuria

## Abstract

*Leishmania (L.) infantum* infections in dogs can cause severe recurrent disease. The aim of this study was to investigate different parameters for early detection of disease relapses in *L. infantum*-infected dogs in Germany. Fifty-two dogs naturally infected with *L. infantum* were enrolled. During the one-year study period, all dogs remained outside of endemic areas and attended study appointments every three months, including physical examination, blood pressure measurement, complete blood count with differential, serum biochemistry with symmetrical dimethylarginine and C-reactive protein, complete urinalysis including urine protein-to-creatinine ratio, *L. infantum* PCR, and antibody ELISA. Disease relapse was defined as deterioration of clinical or laboratory parameters in dogs that had achieved complete or partial remission before. Univariable and multivariable Bayesian logistic regression were used to identify predictors of disease relapse. Lymphadenopathy (*p* < 0.01; OR = 6.93), seborrhea/hypotrichosis (*p* = 0.02; OR = 8.02), and proteinuria (*p* < 0.01; OR = 9.14) were significantly associated with upcoming disease relapses (*n* = 10; 9/52 dogs), while associations between higher antibody levels and upcoming disease relapses trended towards significance (*p* = 0.06; OR = 1.03). Different parameters are important for an early diagnosis of disease relapse in canine leishmaniosis and should thus be regularly assessed and interpreted accordingly in the monitoring of *L. infantum*-infected dogs.

## 1. Introduction

Canine leishmaniosis is the manifestation of *Leishmania (L.) infantum* infection in dogs and occurs endemically in different countries all over the world [1]. The endemicity of the disease is linked to the occurrence of phlebotomine sandflies, which serve as the protozoan parasites’ vector [2,3,4,5]. Manifestation of the disease is commonly linked to an exuberant humoral immune response to the intracellular pathogen, with an excessive production of non-protective antibodies [6,7,8,9]. Together with free *Leishmania* antigens, antibodies form circulating immune complexes. Tissue deposition of immune complexes is one of the main pathological mechanisms underlying signs of canine leishmaniosis, besides other inflammatory and immune-mediated reactions [10,11,12]. Various organ systems can be affected; therefore, a variety of clinical signs and/or laboratory alterations can emerge, even several years after infection [10,13,14]. Thus, close monitoring of infected dogs is considered essential for early recognition of new signs and implementation of appropriate countermeasures [15,16].

To relieve the signs of disease and reduce the parasitic burden, different therapeutic interventions are applied [17,18,19,20]. Commonly, the l leishmanistatic drug allopurinol is used, either alone or in combination with and/or following leishmanicidal treatment with miltefosine or meglumine antimoniate [15]. Since dogs serve as reservoir hosts for *L. infantum*, infections usually cannot be cleared [21,22,23,24], and the re-emergence of disease signs after an initial improvement in response to treatment is common [25,26].

Thus, disease relapses, i.e., their prevention, diagnosis, and treatment, are an important aspect in the management of canine leishmaniosis [27,28,29,30,31,32]. Nevertheless, studies on this topic are scarce. In endemic countries, it is often difficult to differentiate relapse from re- or superinfection. Therefore, studies on relapse in non-endemic countries are highly beneficial. Thus, the aim of this prospective longitudinal study, in which dogs were monitored over a one-year observation period with predefined follow-up appointments, was to evaluate various clinical and laboratory parameters of canine leishmaniosis for their use as predictors of disease relapses in *L. infantum*-infected dogs living in a non-endemic country.

## 2. Materials and Methods

### 2.1. Study Design

This prospective longitudinal clinical study was authorized by the ethical committee of the Centre for Clinical Veterinary Medicine of the LMU Munich (Government of Upper Bavaria, reference number 244-06-12-2020) and was conducted between 2021 and 2023.

#### 2.1.1. Enrollment

Prior to enrollment, the following inclusion criteria had to be fulfilled: (1) proof of *L. infantum* infection, diagnosed by the referring veterinarian, based on a positive PCR (at an external laboratory) or antibody ELISA or IFAT result (according to the respective laboratories’ cut-off values) (Supplementary Table S1); (2) indication of antileishmanial treatment [15]; (3) agreement of dog owners to attend study appointments every three months and not to take their dogs to endemic countries during the observation period. Dogs were excluded from enrollment in case of (1) systemic immunosuppressive treatment or domperidone, (2) severe concomitant disease, or (3) untreated *Ehrlichia (E.) canis* or *Dirofilaria (D.) immitis* co-infections.

Investigations at the time point of enrollment (study day 0) included a qualitative point-of-care (POC) test for *E. canis*, *D. immitis*, *Borrelia burgdorferi,* and *Anaplasma phagocytophilum/platys* (SNAP^®^ 4Dx Plus, IDEXX Laboratories Inc., Westbrook, ME, USA) and an abdominal ultrasound screening to exclude (severe) concomitant diseases and untreated *E. canis* or *D. immitis* co-infections. The enrollment further included a physical examination, non-invasive blood pressure measurement, complete blood count, serum biochemistry including symmetric dimethylarginine (SDMA) and C-reactive protein (CRP), complete urinalysis including urine protein-to-creatinine ratio (UPC) *L. infantum* antibody ELISA, and quantitative PCR of conjunctival swabs. Conjunctival swab samples were collected from both eyes by rubbing a sterile cotton swab along the conjunctival surface.

During the study course, each dog received treatment according to its individual clinical signs. Antileishmanial treatment was applied in standard dosing regimens described in the current literature [15,21,33,34], including allopurinol at 10 mg/kg q12h PO (consideration of dose reduction in case of urinary tract adverse events (Supplementary Figure S1)) and, when indicated, miltefosine at 2 mg/kg q24h PO for 4 weeks or meglumine antimoniate at 100 mg/kg q24h SC for 4 weeks. A low-purine diet was recommended for the prevention of xanthine urolithiasis [33,35]. Supportive symptomatic treatment was applied as needed.

A total of 52 dogs infected with *L. infantum* were enrolled (Supplementary Table S1). The dogs were more often female (*n* = 32) than male (*n* = 20) and mixed breed (*n* = 36) than purebred (*n* = 16), with ages ranging between 11 months and 14 years. All dogs had a travel history or originated from an endemic country. Regarding their *L. infantum* infections prior to inclusion, the dogs either (1) never had severe signs of the disease (*n* = 17), (2) had achieved (*n* = 16) or had not (yet) achieved (*n* = 12) remission by treatment after previous disease manifestation, or (3) had signs of the disease but had not received treatment before (*n* = 7). At enrollment, 40 dogs presented with mild to moderate disease (LeishVet stage I (*n* = 33) or stage II (substage IIa: *n* = 6; substage IIb: *n* = 1)), whereas 12 dogs were classified as severely affected (LeishVet stage III (*n* = 9) or stage IV (*n* = 3)) [36].

#### 2.1.2. Study Appointments

**Figure 1:**
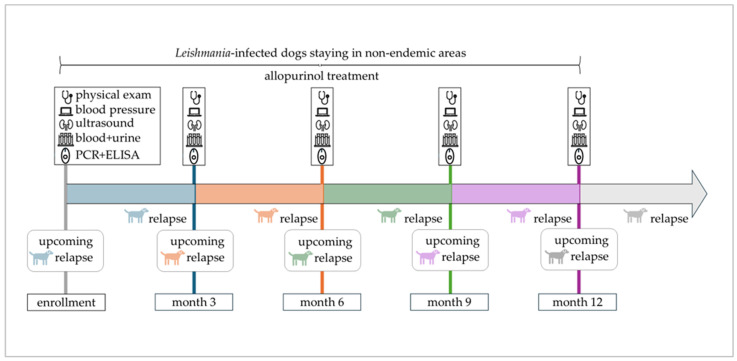
Timeline of the one-year study period with scheduled study appointments. Enrolled dogs remained in non-endemic areas, were treated with allopurinol, and attended study appointments every three months. An upcoming relapse indicates the emergence of disease relapse within 3 months after a scheduled study appointment.

After enrollment, the one-year study period included four appointments at three-month intervals at the LMU Small Animal Clinic in Munich (Figure 1). Each appointment consisted of a thorough physical examination, non-invasive blood pressure measurement, ultrasound of the urinary tract, complete blood count, serum biochemistry including SDMA and CRP, complete urinalysis including UPC, *L. infantum* antibody ELISA, and quantitative PCR (as described for enrollment) of conjunctival swabs.

#### 2.1.3. Laboratory Methods and Instruments Used

For every study appointment, hematological analysis was performed with an automated in-house analyzer (Sysmex XT-2000iV; Sysmex Corporation, Kobe, Japan, or ProCyte Dx, IDEXX Laboratories Inc., Westbrook, ME, USA). Urine specific gravity was determined by an optical refractometer, and urine dipstick analysis was performed using IDEXX UA test strips read by the IDEXX UA Analyzer (IDEXX Laboratories Inc., Westbrook, ME, USA). For urine sediment analysis, an automated in-house device was used (SediVue, IDEXX Laboratories Inc., Westbrook, ME, USA).

Blood biochemistry, UPC, *L. infantum* antibody, and PCR were evaluated in an external laboratory (IDEXX GmbH, Kornwestheim, Germany). *Leishmania* spp. DNA was detected by a validated real-time PCR (IDEXX Laboratories) targeting the glycoprotein gp63 gene as previously described [37]. The assay included quantitative PCR-positive and PCR-negative controls, negative extraction controls, a quantitative internal DNA quality control f (host 18S rRNA), a positive internal control (added to the lysis solution), and a monitoring control for environmental contamination [37]. For the sample shipment, which was performed on the day of sampling, cooled aliquots of urine and serum, as well as conjunctival swabs, were packed in an isolated container. Any surplus material was stored at −80 °C.

### 2.2. Disease Relapse

Disease relapse was defined as either (1) appearance of new clinical or clinicopathological abnormalities, or (2) reappearance of clinical or clinicopathological abnormalities that had been present but had been cleared, or (3) worsening of clinical or clinicopathological abnormalities that had been stable or had improved previously. This applied to dogs that achieved complete remission (restoration of all clinical and laboratory parameters) or partial remission (improvement but no complete restoration of all clinical and laboratory parameters) following previous therapeutic intervention. Differential diagnoses were ruled out whenever dogs presented with new or worsening signs. To identify predictors for disease relapse, clinical and laboratory parameters at the study appointment preceding the relapse within the following three months were considered (Figure 1). Accordingly, disease relapses observed at the first study appointment were not included in predictor analysis due to missing preceding data.

### 2.3. Statistical Analysis

Statistical analysis was performed using R statistical software (version 4.3.1). All study appointments were treated as independent events. If dogs were withdrawn from the study before completion of the observation period, data obtained up to the time of exclusion were included in the analyses.

Associations between upcoming disease relapses and clinical signs, as well as laboratory alterations, were analyzed by univariable and multivariable logistic Bayesian regression. After univariable analysis, parameters with *p*-values < 0.2 were included in the corresponding multivariable model [38]. Model selection was performed by an automated model selection and multimodel inference (brute-force) approach using the R package “glmulti” [39,40]. Model performance was evaluated by the corrected Akaike Information Criterion (AICc); models with the lowest AICc were selected, and only the remaining predictors in the final multivariable models were analyzed. Statistical significance was defined as *p* ≤ 0.05, with 0.05 < *p* ≤ 0.1 indicating a trend towards significance.

## 3. Results

### 3.1. Study Course

**Figure 2:**
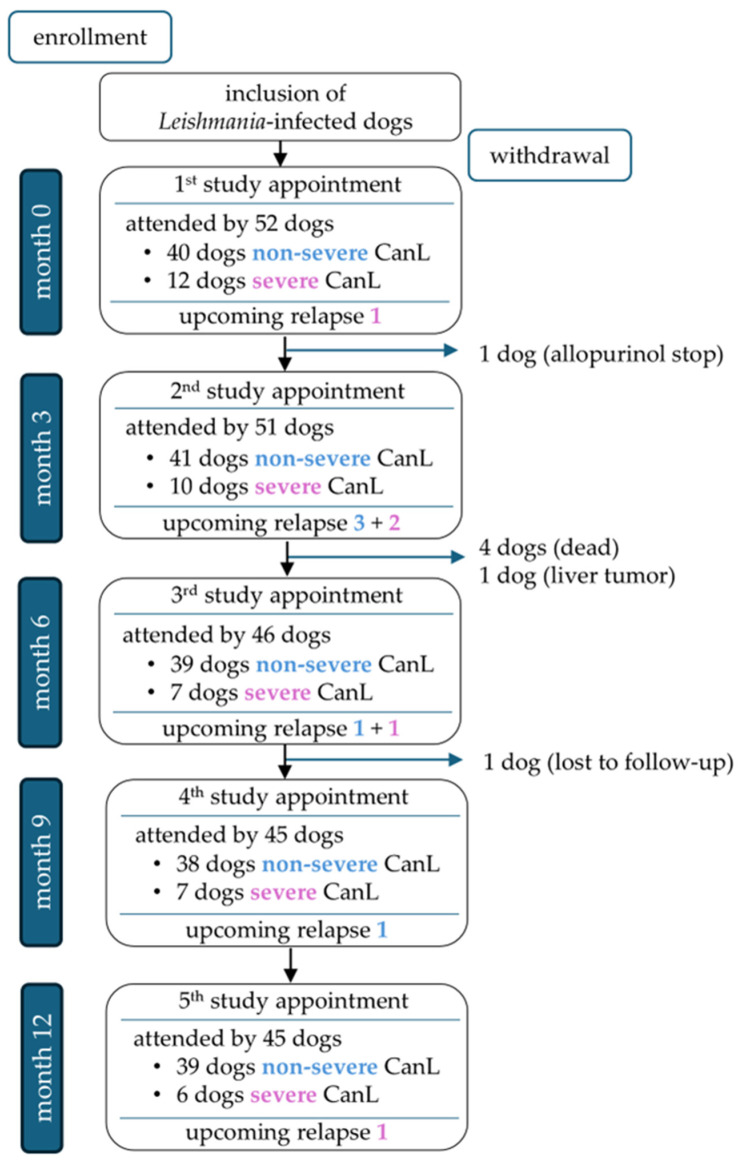
Overview of dogs attending the study appointments at months 0, 3, 6, 9, and 12, divided into non-severe cases (blue font; LeishVet stages I–II) and severe cases (pink font; LeishVet stages III–IV) [33], including upcoming relapses per group.

A total of 45/52 dogs completed the one-year study period (Figure 2). Seven of 52 dogs were unable to complete the study for the following reasons: One dog died during a disease relapse that was associated with severe non-regenerative anemia and azotemia and occurred approximately 4.5 months after an initial improvement following leishmanicidal and supportive symptomatic treatment. One dog was euthanized with progressive chronic kidney disease, and one dog was diagnosed with a liver tumor. Two dogs died acutely due to unclarified underlying causes. Another dog was withdrawn from the study due to discontinuation of allopurinol treatment, and one dog was lost to follow-up. Overall, 177 appointments were eligible for statistical analysis of relapse predictors; 10/177 study appointments of 9/52 dogs were followed by disease relapses after 1.5–3 (median 2.4) months (Supplementary Table S2 and Figure S2).

### 3.2. Predictors of Disease Relapse

Various clinical and laboratory parameters were evaluated as predictors of emerging disease relapses (Table 1). Univariable logistic regression (UVA) of clinical signs identified lymphadenopathy (*p* < 0.01), seborrhea/hypotrichosis (*p* < 0.01), and skin ulcers (*p* = 0.04) as significant predictors of upcoming relapse. Based on a preselection threshold of *p* < 0.2, papules/nodules (*p* = 0.08) were also included in the multivariable model, whereas conjunctivitis/blepharitis (*p* = 0.44) was excluded from further analysis. In multivariable logistic regression, lymphadenopathy (OR = 6.93; 95% CI: 1.80–26.7; *p* < 0.01) and seborrhea/hypotrichosis (OR = 8.02; 95% CI: 1.43–44.9; *p* = 0.02) remained significant predictors. Specifically, patients with lymphadenopathy had 6.93-fold increased odds of upcoming relapse compared to those without. Similarly, patients with seborrhea/hypotrichosis had 8.02 times higher odds of upcoming relapse. Papules/nodules and skin ulcers were eliminated from the final model during automated model selection based on the corrected Akaike Information Criterion (AICc).

In terms of laboratory alterations, UVA identified proteinuria (*p* < 0.01), *Leishmania* antibody levels (*p* = 0.05), and hypoalbuminemia (*p* = 0.05) as significant predictors of upcoming relapse. In addition, hyperproteinemia (*p* = 0.06), hyperglobulinemia (*p* = 0.07), monocytosis (*p* = 0.06), and non-regenerative anemia (*p* = 0.17) were included in the multivariable analysis (*p* < 0.2). Thrombocytopenia, neutropenia, lymphopenia, lymphocytosis, renal azotemia, and increased levels of SDMA and CRP were excluded from further analysis (*p* > 0.2). In multivariable logistic regression, proteinuria (OR = 9.14; 95% CI: 2.41–34.6; *p* < 0.01) remained the only significant predictor of upcoming relapse. Specifically, patients with proteinuria had 9.14-fold increased odds of upcoming relapse compared to those without. Associations between *Leishmania* antibody levels and upcoming relapse trended towards significance (OR = 1.03; 95% CI: 1.00–1.05; *p* = 0.06). Hypoalbuminemia, hyperproteinemia, hyperglobulinemia, monocytosis, and non-regenerative anemia were eliminated from the final model during automated model selection based on the corrected Akaike Information Criterion (AICc).

## 4. Discussion

To the best of the authors’ knowledge, this is the first prospective study investigating prognostic parameters for upcoming disease relapses in dogs with *L. infantum* infections in non-endemic countries. With a prevalence of 17% (9/52 dogs), the emergence of disease relapses evaluated in the present study population was comparable to a previous study in an endemic area, with approximately 20% of dogs experiencing relapse, although within a two-year period [26]. However, overall relapse rates vary widely in different previous studies and were reported to even exceed 70% [16], but the extent to which re- or superinfection contributes to worsening and relapse diagnosis in dogs in endemic areas remains unclear [14]. Nevertheless, it points to the relevance of a close monitoring of *L. infantum*-infected dogs and the need for long-term studies performed in non-endemic areas, without risk of re-exposure to the pathogen. To ensure timely therapeutic interventions and counteract an upcoming deterioration, a proper assessment of disease relapse is essential but lacks standardization [27]. According to a survey among 155 veterinarians in Portugal, the majority (approximately 60%) always considered the reappearance or worsening of clinical signs to establish a diagnosis of relapse, while only a minor percentage of veterinarians always considered laboratory parameters in this context [27]. The relevance of laboratory findings for relapse diagnosis was, however, emphasized by a recent retrospective study; besides higher total clinical scores, anemia, dysproteinemia, and high antibody titers (IFAT) were significantly associated with an increased risk of disease relapses in naturally infected dogs in Spain [41]. To account for the gradual disease progression, which is commonly observed rather than a sudden onset [14,42,43,44], the present study evaluated different clinical and laboratory parameters for their use as predictors of disease relapse. Despite the longitudinal study design over a one-year period, the number of dogs with relapse was relatively low (*n* = 9), which might have influenced statistical results.

With regard to the present study’s findings on clinical signs, dogs with lymphadenopathy were found to be significantly (approximately seven times) more likely to experience disease relapse in the following three months than dogs without. Indeed, lymphadenopathy was also identified as the earliest clinical sign emerging in experimentally infected dogs transitioning to overt disease [42,43], which, according to the findings of the present study, might also apply to disease relapse.

Although not pathognomonic for canine leishmaniosis, enlargement of lymph nodes is one of the most common clinical signs of canine leishmaniosis [45,46,47,48] and was even identified as a predictive factor for diagnosing the disease in 713 dogs examined as part of a cross-sectional study performed in a veterinary teaching hospital in Brazil [30]. Accordingly, lymphadenopathy was shown to be a clinical sign prompting suspicion of canine leishmaniosis among veterinarians in endemic countries [49]. Histopathologically, lymph node enlargement has been linked to indirect and direct responses to *L. infantum* infections. These included hyperplasia of lymphoid follicles related to the proliferation of antibody-producing B-cells [50,51] and/or enhanced proliferation of macrophages [50,52,53] and granulomatous inflammation, which is assumed to occur at sites of parasitic infections with a proliferation and/or infiltration of histiocytes, plasma cells, lymphocytes, and in some cases neutrophils and eosinophils [6,12]. Thus, it seems reasonable that disease relapse, which is assumed to result from enhanced parasite replication and/or a shift towards a Th2-predominated immune response [24,25,31,44], is reflected early in lymph node enlargement.

Besides lymphadenopathy, dogs presenting with seborrhea and/or hypotrichosis in the present study were found to have an approximately eight times higher likelihood of upcoming disease relapses than dogs without. In canine leishmaniosis, seborrhea is variably associated with hypotrichosis (to alopecia) and commonly attributable to exfoliative dermatitis [8], presumed to emerge through hematogenous spread of parasites [12,54,55]. Histopathologically, exfoliative dermatitis can be characterized by (interstitial, perivascular, nodular, or periadnexal) granulomatous inflammation including infiltrates of macrophages, lymphocytes, and plasma cells, accompanied by orthokeratotic hyperkeratosis of the epidermis and follicles [12,54,56,57,58]. Since this inflammatory pattern could also be observed in macroscopically non-lesioned skin of *Leishmania*-infected dogs [55,58,59], microscopic alterations might precede (or persist without) macroscopic lesions [55,56,58,60,61]. It could thus be assumed that in case of an emerging disease relapse, an uncontrolled parasite replication leads to dermal inflammation, the macroscopic manifestation of which might be an early indicator of the relapse-associated immunological shift towards a Th2-predominated immune response.

Notably, seborrhea and hypotrichosis/alopecia seem to already constitute a relevant parameter in the clinical monitoring of dogs suspected to be infected with *Leishmania* spp. At least in endemic European countries, exfoliation and alopecia were reported by veterinarians as signs suggestive of canine leishmaniosis [49]. Furthermore, exfoliation and alopecia were shown to be associated with the diagnosis of *L. infantum* infections in high-prevalence areas in Spain [62]. In Brazil, periorbital alopecia was identified as a clinical sign significantly associated with canine leishmaniosis in dogs [30]. Thus, as indicated by the present results, seborrhea and/or hypotrichosis/alopecia are of prognostic relevance not only for diagnosing the disease but also for disease relapse.

Regarding laboratory alterations, proteinuria was the only significant predictor of disease relapse in the present study. Proteinuria is estimated to affect approximately 50% of naturally *L. infantum*-infected dogs [17,28,33,45]; it occurs primarily due to immune complex deposition and the associated inflammatory immune response in glomeruli [63] and can cause renal failure, the most common cause of death in canine leishmaniosis [10,29,33,64]. Previous studies have proved proteinuria in dogs with leishmaniosis to be linked to poor prognosis and reduced survival time [17,28,65]. Thus, the onset of adequate therapeutic measures, which commonly include antileishmanial and symptomatic antiproteinuric treatment, is considered essential to counteract proteinuria in dogs with leishmaniosis [33,66]. To counteract the immunopathogenic aspect of glomerulonephritis, i.e., lower the inflammatory response to immune complex deposition, the (additional) use of immunosuppressive drugs is frequently discussed and applied by some veterinarians [33,67]. In general, treatment of glomerulonephritis is considered successful if UPC decreases to ratios below 0.5 or by at least 50% [66]. However, in case of irreversible renal damage, proteinuria might persist despite any treatment. Persistent proteinuria was also observed in several dogs in the present study and should be taken into account when interpreting the results, particularly with regard to upcoming disease relapse. Nevertheless, given that proteinuria represents a risk factor for the progression of renal impairment and is also linked to a higher probability of disease relapse, early recognition of any deterioration in UPC ratios must be ensured through routine assessment of UPC ratios at each monitoring appointment. In a study on apparently healthy dogs infected with *L. infantum,* 31/87 dogs (35.6%) were found to have proteinuria; therefore, awareness of this topic is important [9]. Of concern, however, is the fact that the relevance of proteinuria for disease and relapse prognosis might still be neglected in clinical practice; according to a questionnaire-based study among veterinarians in Portugal, less than 30% of all respondents always assessed UPC in the monitoring of dogs with canine leishmaniosis and commonly did not even prioritize it when caregivers were financially restricted [27]. To overcome this diagnostic gap, regular urine dipstick monitoring by either the owners at home or the veterinarian might offer a cost-effective alternative. It was proven to be a reliable, sensitive, although not specific, method to assess urinary protein loss in dogs, even if results might be influenced by urine specific gravity and active sediment. Due to low specificity, a protein loss indicated by a urine dipstick should always be confirmed by complete urinalysis and UPC measurement [64,68].

In the present study, higher *Leishmania* antibody levels trended towards a significant association with upcoming disease relapses. In previous studies, increasing antibody titers were shown to be associated with disease relapse [25,41]. Accordingly, the monitoring of antibody titers and suspicion of disease relapse in case of a marked (more than twofold) increase is suggested by current guidelines [15] and is implemented by (some) veterinarians, at least in endemic countries; in a questionnaire-based study in Portugal, approximately 25% of the veterinarians that responded always considered increasing antibody titers for relapse diagnosis [27]. However, findings on antibodies in dogs in endemic areas might not be directly comparable to studies performed in non-endemic countries, since seasonal variations in antibody titers without clinical relevance, possibly related to a re-exposure to the pathogen, were observed in dogs living in endemic areas [69,70]. Inconclusive findings also exist on the prognostic relevance of antibody measurement. Commonly, high antibody levels are observed in dogs in severe disease stages, which are estimated to have an overall guarded to poor prognosis [16,71,72]. This is attributed to the fact that dogs presenting with high antibody titers mount a Th2-dominated immune response, which lacks protective effects against intracellular parasite replication and favors the formation of immune complexes, thereby promoting the development of disease signs [6,11]. Nevertheless, antibody titers were not associated with survival time of dogs with canine leishmaniosis in a retrospective study in a non-endemic country [17]. Overall, interpretation of antibody titers might also be complicated by previous therapeutic measures; high antibody titers were shown to persist for up to six months or even longer after treatment, despite clinical improvement [15,16,64,73]. In conclusion, increasing and/or high antibody titers might need to be interpreted cautiously in pre-treated dogs (and should not justify the initiation of leishmanicidal treatment on their own, at least in non-endemic countries).

Levels of acute phase proteins are discussed to provide information on disease severity in dogs with leishmaniosis and are thus often included in the monitoring of dogs with leishmaniosis [64,74]. However, high levels of acute-phase proteins were not identified as predictors for upcoming disease relapse in the present study.

Furthermore, results of *Leishmania* PCR (qualitative and quantitative) have been proposed as a tool for monitoring and assessment of dogs infected with *Leishmania* spp. [61,64,75,76], but an association with upcoming relapse could not be evaluated in the present study due to a surprisingly low number of PCR-positive conjunctival swabs. A previous study conducted in dogs living in a non-endemic country revealed an adequate sensitivity (78.4%) of conjunctival swab PCR and substantial to moderate correlation with lymph node and bone marrow PCR [77]; variations in applied PCR techniques could have contributed to the observed discrepancy between the aforementioned and the present study’s result. Further studies on diagnostic and prognostic values of positive PCR results for disease relapse, including other low-invasively collected specimens such as vulvar and oral swabs [42,78] and/or different tissues, e.g., lymph nodes [79,80], would be valuable.

One limitation of the present study was the overall low number of dogs that experienced disease relapse during the study period. In addition, none of the dogs that died underwent necropsy, and thus, an association with *Leishmania* infection and potentially missed disease relapses cannot be ruled out with certainty.

## 5. Conclusions

Lymphadenopathy, seborrhea, and/or hypotrichosis, as well as proteinuria, were identified as predictors for relapse of disease in the present study on *L. infantum*-infected dogs in a non-endemic country. In addition, associations might exist with antibody titers measured by ELISA, but subsequent studies are necessary to evaluate their prognostic relevance. The findings emphasize the routine monitoring and the importance of including these predictors as part of routine monitoring in dogs with *L. infantum* infections to ensure an adequate follow-up, an early recognition of disease relapse, and concomitantly the onset of adequate therapeutic measures to prevent deterioration.

## Figures and Tables

**Table 1:** Univariable and multivariable logistic Bayesian regression of clinical and laboratory parameters as predictors of upcoming disease relapses in *Leishmania*-infected dogs in Germany.

Clinical Parameter			Upcoming Relapse								UVA	MVA		
			Yes (*n* = 10)				No (*n* = 167)				*p*	OR	95%CI	*p*
			*n*	Med	Range		*n*	Med	Range					
clinical signs	lymphadenopathy	yes	7				26				<0.01	6.93	1.80–26.7	**<0.01**
		no	3				141							
	seborrhea/ hypotrichosis	yes	9				57				<0.01	8.02	1.43–44.9	**0.02**
		no	1				110							
	papules/nodules	yes	2				7				0.08	removed (AICc)		
		no	8				160							
	skin ulcers	yes	2				5				0.04	removed (AICc)		
		no	8				162							
	conjunctivitis/ blepharitis	yes	1				6				0.44	n.a.		
		no	9				161							
**Laboratory Parameter**														
complete blood count	non-reg. anemia *hct < 35/37.3% (H1/H2)*	yes	1	*31.8*	*-*	*-*	2	*27.4*	*21.2*	*33.5*	0.17	removed (AICc)		
		no	9	*42.3*	*35.5*	*49.2*	165	*47.1*	*34.0*	*60.6*				
	thrombocytopenia *plt < 150/148 × 10^9^/L (H1/H2)*	yes	1	*121*	*-*	*-*	6	*143*	*112*	*149*	0.44	n.a.		
		no	9	*278*	*201*	*791*	161	*242*	*154*	*624*				
	neutropenia *neut < 3.00/2.95 × 10^9^/L (H1/H2)*	yes	0	*-*	*-*	*-*	15	*2.43*	*2.08*	*2.97*	0.39	n.a.		
		no	10	*4.4*	*3.01*	*8.28*	152	*4.43*	*2.98*	*10.2*				
	lymphopenia *lymph < 1.00/1.05 × 10^9^/L (H1/H2)*	yes	1	*0.99*	*-*	*-*	8	*0.83*	*0.69*	*0.99*	0.56	n.a.		
		no	9	*1.89*	*1.05*	*2.75*	159	*2.13*	*1.00*	*4.16*				
	lymphocytosis *lymph > 3.6/5.1 × 10^9^/L (H1/H2)*	yes	0	*-*	*-*	*-*	8	*4.1*	*3.63*	*4.16*	0.57	n.a.		
		no	10	*1.62*	*0.99*	*2.75*	159	*2.02*	*0.69*	*3.51*				
	monocytosis *mono > 0.5/1.12 × 10^9^/L (H1/H2)*	yes	3	*0.63*	*0.55*	*1.17*	14	*0.59*	*0.51*	*0.75*	0.06	removed (AICc)		
		no	7	*0.19*	*0.13*	*0.48*	153	*0.28*	*0.11*	*0.5*				
serum biochemistry	hypalbuminemia *alb < 2.8 g/dL*	yes	5	*2.4*	*1.7*	*2.7*	33	*2.6*	*1.6*	*2.7*	0.05	removed (AICc)		
		no	5	*3.2*	*2.8*	*3.7*	134	*3.1*	*2.8*	*3.7*				
	hyperproteinemia *tp > 7.6 g/dL*	yes	2	*8.1*	*7.7*	*8.4*	6	*8.3*	*7.7*	*9.7*	0.06	removed (AICc)		
		no	8	*6.7*	*6.0*	*7.5*	161	*6.5*	*5.5*	*7.5*				
	hyperglobulinemia *glob > 4.3 g/dL*	yes	3	*4.6*	*4.4*	*6.6*	15	*4.7*	*4.4*	*7.0*	0.07	removed (AICc)		
		no	7	*4.0*	*3.2*	*4.3*	152	*3.4*	*2.5*	*4.3*				
	CRP increased *CRP > 10.7 mg/L*	yes	1	*31.9*	*-*	*-*	14	*17.6*	*11.0*	*31.2*	0.88	n.a.		
		no	9	*4.2*	*3.0*	*9.6*	153	*3.3*	*0.5*	*10.6*				
	SDMA increased *SDMA > 14 μg/dL*	yes	2	*25*	*23*	*26*	33	*16*	*15*	*37*	0.88	n.a.		
		no	7	*12*	*9*	*14*	133	*12*	*2*	*14*				
	renal azotemia *crea ≥ 1.4 mg/dL*	yes	2	*1.9*	*1.8*	*1.9*	12	*1.6*	*1.4*	*2.2*	0.22	n.a		
		no	8	*0.9*	*0.3*	*1.0*	155	*0.8*	*0.5*	*1.3 * ^a^				
others	proteinuria UPC > 0.5 *	yes	5	*4.2*	*0.8*	*12.6*	13	*1.0*	*0.7*	*3.0*	<0.01	9.14	2.41–34.6	**<0.01**
		no	5	*0.1*	*0.1*	*0.3*	*0.1*	*0.1*	*0.5*	*0.1*				
	antibody level *TU > 12*	TU	10	*39.1*	*4.1*	*88.3*	167	*20.6*	*0.1*	*110.5*	0.05	1.03	1.00–1.05	0.06

Parameters with *p* < 0.2 in univariable analyses (UVA) were included in multivariable analyses (MVA); model selection was based on an automated model selection and multimodel inference approach, ranked based on the corrected Akaike Information Criterion (AICc). Level of significance: *p* ≤ 0.05; alb, albumin; CI, confidence interval; crea, creatinine; CRP, C-reactive protein; glob, globulin; H1, automated hematological analyzer 1 (=Sysmex XT-2000iV; Sysmex Corporation, Kobe, Japan); H2, automated hematological analyzer 2 (=ProCyte Dx, IDEXX Laboratories Inc., Westbrook, ME, USA); hct, hematocrit; lymph, lymphocytes; mono, monocytes; med, median; *n*, number of dogs per category; n.a., not applicable; neut, neutrophils; OR, odds ratio; plt, platelets; SDMA, symmetric dimethylarginine; tp, total protein; TU, test unit (ELISA); UPC, urine protein-to-creatinine; *, censored in case of 0.5 < UPC < 2.0 and active sediment (>5 leukocytes and/or epithelial cells per high power field, bacteria and/or spermatozoa) or macroscopic hematuria; ^a^, not listed if increased without renal azotemia.

## Data Availability

The raw data supporting the conclusions of this article will be made available by the authors on request.

## Supplementary Materials

The following supporting information can be downloaded at: https://www.mdpi.com/article/10.3390/pathogens14121282/s1, Table S1: Baseline characteristics of dogs before and at the time of enrollment; Table S2: Disease parameters of dogs with relapse of canine leishmaniosis during the one-year study period; Figure S1: Treatment of the 52 dogs included in the study guided by the therapeutic tree for dogs with Leishmania infections in non-endemic areas according to Kaempfle et al., 2025 [21]; Figure S2: Occurrence of upcoming relapse of canine leishmaniosis in 52 dogs during the one-year study period.
