# Supplementary material for: Importance of Different Parameters for Monitoring Dogs with Leishmania infantum Infections in a Non-Endemic Country
Source: Pathogens. 2025 Dec 12;14(12):1282. doi: 10.3390/pathogens14121282 (PMC12735808; doi:10.3390/pathogens14121282)
Supplement: Supplementary file 1 [file pathogens-14-01282-s001.zip › Supplementary Table S1.pdf]

Supplementary Table S1. Baseline characteristics of dogs before and at the time of enrollment

Dog	Disease parameters of CanL before enrollment			Inclusion criteria		Disease parameters of CanL at time of enrollment		
No.	previous clinical signs*	previous laboratory alterations*	previous causal treatment	ELISA/ IFAT	ref	clinical signs	laboratory alterations	ELISA[c]
1	skin ulcers, seborrhea/hypotrichosis	anemia, hyperproteinemia, hypalbuminemia, proteinuria	miltefosine, allopurinol	116[a]	≤12	-	-	43.1
2	seborrhea/hypotrichosis, papules/nodules, skin ulcers, uveitis	anemia, thrombocytopenia, hyperproteinemia, hypalbuminemia, hyperglobulinemia	miltefosine, meglumine antimoniate, allopurinol	1:1000[b]	≤1:100	skin ulcers, uveitis, seborrhea/hypotrichosis, lymphadenopathy	anemia, hypalbuminemia, hyperproteinemia, hyperglobulinemia	59.6
3	seborrhea/hypotrichosis	anemia, thrombocytopenia, hyperproteinemia, hypalbuminemia hyperglobulinemia	miltefosine, allopurinol	72.2[a]	≤12	mild seborrhea/hypotrichosis	-	21.8
4	conjunctivitis	anemia, leukopenia/neutropenia	-	1:500[b]	≤1:100	conjunctivitis	-	0.1
5	lameness	anemia, hyperproteinemia, hypalbuminemia, proteinuria	allopurinol	82.3[a]	≤12	mild seborrhea/hypotrichosis	anemia, thrombocytopenia, lymphopenia, hypalbuminemia, hyperglobulinemia, hyperproteinemia, proteinuria	44.9
6	seborrhea/hypotrichosis, lymphadenopathy, skin ulcers, papules/nodules	hypalbuminemia	miltefosine, allopurinol	46.6[a]	≤12	papules/nodules	-	24.9
7	seborrhea/hypotrichosis, onychogryphosis	hypalbuminemia, azotemia, proteinuria	miltefosine, allopurinol	32.2[a]	≤12	-	hypalbuminemia, azotemia, proteinuria	26.3
8	skin ulcers, seborrhea/hypotrichosis	hyperglobulinemia, hypalbuminemia	allopurinol	50.9[a]	≤12	mild seborrhea/hypotrichosis	lymphopenia	3.1
9	seborrhea/hypotrichosis, papules/nodules	-	allopurinol	66.8[a]	≤12	mild seborrhea/hypotrichosis	-	51.2
10	seborrhea/hypotrichosis, skin ulcers	leukopenia with neutropenia, hyperproteinemia, hypalbuminemia hyperglobulinemia, proteinuria	meglumine antimoniate, allopurinol	2.91[a]	≤1.1	mild seborrhea/hypotrichosis	proteinuria	5.4
11	seborrhea/hypotrichosis	-	allopurinol	2.36[a]	≤1.1	-	hyperglobulinemia, hyperproteinemia	11.3
12	alopecia	-	allopurinol	0.88[a]	≤0.7	-	-	12.4
13	seborrhea/hypotrichosis, skin ulcers, lymphadenopathy, edema	hyperproteinemia, hypalbuminemia, hyperglobulinemia, proteinuria	meglumine antimoniate miltefosine, allopurinol	46.4[a]	≤12	seborrhea/hypotrichosis	hypalbuminemia	26.1
14	seborrhea/hypotrichosis, lymphadenopathy, skin ulcers, lameness	-	miltefosine, allopurinol	3.07[a]	<0.8	-	-	5.0
15	lymphadenopathy	hypalbuminemia, azotemia, proteinuria	miltefosine, allopurinol	82.5[a]	≤12	lymphadenopathy, seborrhea/hypotrichosis	monocytosis, hypalbuminemia, azotemia, proteinuria	69.2

16	epistaxis	anemia, azotemia, hypalbuminemia, proteinuria	meglumine antimoniate, allopurinol	49.5[a]	≤12	-	-	2.1
17	apathy	pancytopenia	miltefosine, meglumine antimoniate, allopurinol	45.6[a]	≤12	-	-	16.0
18	papules/nodules, seborrhea/hypotrichosis	-	allopurinol	67.8[a]	≤12	mild seborrhea/hypotrichosis, papules/nodules	-	67.6
19	papules/nodules, seborrhea/hypotrichosis	hyperglobulinemia	miltefosine, allopurinol	14.9[a]	≤12	-	-	8.3
20	lymphadenopathy, skin lesions	anemia, hyperproteinemia, hypalbuminemia, proteinuria	meglumine antimoniate, allopurinol	3.36[a]	<0.8	mild seborrhea/hypotrichosis. lymphadenopathy	monocytosis, hypalbuminemia, proteinuria	40.9
21	seborrhea/hypotrichosis, skin ulcers	anemia, neutropenia	miltefosine, allopurinol	40.1[a]	≤12	-	-	40.7
22	seborrhea/hypotrichosis	anemia, hypalbuminemia, hyperglobulinemia	miltefosine, allopurinol	20.2[a]	≤12	-	-	17.6
23	-	hypalbuminemia, azotemia	allopurinol	2.72[a]	≤1.1	-	mild azotemia	21.6
24	seborrhea/hypotrichosis	hyperproteinemia, hyperglobulinemia	allopurinol	36.9[a]	≤12	mild seborrhea/hypotrichosis	hyperglobulinemia, hyperproteinemia	29.7
25	-	anemia, leukopenia with neutropenia and lymphopenia, hypalbuminemia, hyperglobulinemia	miltefosine, allopurinol	41.4[a]	≤12	-	hypalbuminemia	4.1
26	-	anemia, leukopenia with neutropenia/lymphopenia, thrombocytopenia, hypalbuminemia, hyperglobulinemia, azotemia, proteinuria	miltefosine, allopurinol	34.2[a]	≤12	-	hypalbuminemia, proteinuria	29.1
27	skin ulcers, papules/nodules	hyperproteinemia	miltefosine, allopurinol	2.88[a]	≤1.1	-	-	43.1
28	-	anemia, hypalbuminemia, hyperglobulinemia	miltefosine, allopurinol	1:400[b]	<1:50	-	-	25.1
29	seborrhea/hypotrichosis	anemia, thrombocytopenia, hypalbuminemia, hyperproteinemia, azotemia, hyperglobulinemia, proteinuria	meglumine antimoniate, miltefosine, allopurinol	42.7[a]	≤12	mild seborrhea/hypotrichosis, lymphadenopathy	anemia, hypalbuminemia, hyperglobulinemia, hyperproteinemia, azotemia, proteinuria	51.8
30	history of litter of diseased puppies	-	allopurinol	1.37[a]	≤1.1	-	-	1.6
31	seborrhea/hypotrichosis, conjunctivitis, papules/nodules, lymphadenopathy, arthritis	anemia, thrombocytopenia, hyperproteinemia, hypalbuminemia	miltefosine, meglumine antimoniate, allopurinol	38.6[a]	≤12	mild seborrhea/hypotrichosis, papules/nodules, lymphadenopathy	-	51.4
32	seborrhea/hypotrichosis,	hyperproteinemia, hyperglobulinemia	allopurinol	57.3[a]	≤12	seborrhea/hypotrichosis	monocytosis	38.3
33	seborrhea/hypotrichosis	anemia	-	4.46[a]	≤1.1	mild seborrhea/hypotrichosis lymphadenopathy	-	31.8

34	seborrhea/hypotrichosis, lameness	hyperproteinemia, hyperglobulinemia, hypalbuminemia	allopurinol	48.3[a]	≤12	-	-	25.9
35	epistaxis, lymphadenopathy	anemia, hyperproteinemia, hypalbuminemia	miltefosine, allopurinol	85.7[a]	≤12	lymphadenopathy	neutropenia	68.4
36	skin ulcer, seborrhea/hypotrichosis	anemia, hypalbuminemia, hyperglobulinemia, hyperproteinemia, proteinuria	miltefosine, meglumine antimoniate, allopurinol	3.91[a]	<0.8	-	-	29.4
37	skin ulcers, seborrhea/hypotrichosis, papules/nodules	hyperproteinemia, hypalbuminemia, hyperglobulinemia, proteinuria	miltefosine, allopurinol	58.6[a]	≤12	skin ulcers, seborrhea/hypotrichosis	monocytosis, hypalbuminemia, hyperglobulinemia, hyperproteinemia, proteinuria	50.9
38	skin ulcer	-	-	58.8[a]	≤12	skin ulcer, mild seborrhea/hypotrichosis	lymphopenia, hypalbuminemia, hyperglobulinemia, proteinuria	40.1
39	ocular lesions	-	allopurinol	32.0[a]	≤12	skin ulcers, lymphadenopathy, conjunctivitis/blepharitis, keratitis/uveitis	monocytosis, hyperglobulinemia	29.6
40	skin ulcer	hyperproteinemia, hypalbuminemia, hyperglobulinemia	allopurinol	28.0[a]	≤12	-	hypalbuminemia	15.0
41	skin ulcer	hypalbuminemia	meglumine antimoniate, allopurinol	29.4[a]	≤12	skin ulcer	hypalbuminemia	23.5
42	fever, apathy	leukopenia with neutropenia and lymphopenia, thrombocytopenia, hyperproteinemia, hypalbuminemia	miltefosine, allopurinol	87.6[a]	≤12	mild seborrhea/hypotrichosis	-	66.7
43	seborrhea/hypotrichosis, skin ulcers, lymphadenopathy	anemia, hyperproteinemia, hyperglobulinemia, hypalbuminemia	miltefosine, meglumine antimoniate, allopurinol	103.9[a]	≤12	seborrhea/hypotrichosis, lymphadenopathy	hyperglobulinemia, hyperproteinemia	110.5
44	lameness	hyperglobulinemia	allopurinol	25.8[a]	≤12	-	-	5.0
45	seborrhea/hypotrichosis, skin ulcer	hyperproteinemia, hyperglobulinemia, hypalbuminemia	miltefosine, allopurinol	72.9[a]	≤12	lymphadenopathy, seborrhea/hypotrichosis	monocytosis, hypalbuminemia, hyperglobulinemia	72.1
46	seborrhea/hypotrichosis, skin ulcer	anemia, hyperglobulinemia, hyperproteinemia	allopurinol	3.13[a]	<0.8	-	anemia, monocytosis, hyperglobulinemia, hyperproteinemia	35.7
47	skin ulcer, seborrhea/hypotrichosis, epistaxis	anemia, thrombocytopenia, leukopenia with neutropenia hyperproteinemia, hypalbuminemia, proteinuria	miltefosine, allopurinol	1:1280[b]	≤1:80	mild seborrhea/hypotrichosis	thrombocytopenia, hypalbuminemia, hyperglobulinemia,	47.5
48	edema, epistaxis	anemia, hypalbuminemia, azotemia, proteinuria	miltefosine, meglumine antimoniate, allopurinol	16.8[a]	≤12	-	thrombocytopenia, neutropenia, hypalbuminemia, azotemia, proteinuria	16.5
49	-	-	allopurinol	31.6[a]	≤12	-	-	10.6
50	skin ulcers, hypotrichosis/seborrhea, lameness	thrombocytopenia, neutropenia, lymphopenia, hyperglobulinemia hyperproteinemia, hypalbuminemia	miltefosine, allopurinol	50.9[a]	≤12	lymphadenopathy, seborrhea/hypotrichosis	neutropenia, hypalbuminemia, hyperglobulinemia, hyperproteinemia	54.1
51	seborrhea/hypotrichosis	-	allopurinol	20.2[a]	≤12	mild seborrhea/hypotrichosis	-	13.5

52	lymphadenopathy, seborrhea/hypotrichosis	anemia, hyperglobulinemia, hypalbuminemia hyperproteinemia, proteinuria	miltefosine, allopurinol	3.54[a]	<0.8	mild seborrhea/hypotrichosis	hypalbuminemia	18.8

*as far as known; CanL, canine leishmaniosis; [a] antibody ELISA result expressed in ELISA units; [b] antibody IFAT result expressed as antibody titer; ref, reference range of the respective laboratory indicating clearly positive antibody results (borderline values were not considered); [c] antibody ELISA performed at IDEXX GmbH, Kornwestheim, Germany throughout the study (results expressed in ELISA test units (TE); results >12 TE were considered positive); colored cells indicate relapse at the first study appointment
