# Supplementary material for: Importance of Different Parameters for Monitoring Dogs with Leishmania infantum Infections in a Non-Endemic Country
Source: Pathogens. 2025 Dec 12;14(12):1282. doi: 10.3390/pathogens14121282 (PMC12735808; doi:10.3390/pathogens14121282)
Supplement: Supplementary file 1 [file pathogens-14-01282-s001.zip › Supplementary Table S2.pdf]

Supplementary Table S2. Disease parameters of dogs with relapse of canine leishmaniosis during the one-year study period

Dog	Study appointment before relapse			Relapse	
No.	month	disease parameters	ELISA (TE)	newly emerging signs	worsening of existing signs
50	3	mild seborrhea/hypotrichosis, mild lymphadenopathy	53.4	skin ulcer, thrombocytopenia, neutropenia, hyperglobulinemia	-
43	6	mild seborrhea/hypotrichosis, papules/nodules, mild lymphadenopathy	88.3	skin ulcers	-
37	6	mild seborrhea/hypotrichosis, proteinuria	30.6	skin ulcers	-
39	3	mild seborrhea/hypotrichosis, mild lymphadenopathy, uveitis, hyperproteinemia, proteinuria	18.9	-	uveitis
25	12	mild hypalbuminemia, azotemia	4.1	anemia, leukopenia with neutropenia	-
20	0	mild seborrhea/hypotrichosis, mild lymphadenopathy, hypalbuminemia, proteinuria	40.9	anemia, neutropenia	proteinuria
29	3	mild seborrhea/hypotrichosis, mild lymphadenopathy, mild anemia/thrombocytopenia, hyperproteinemia, hyperglobulinemia, hypalbuminemia, azotemia, proteinuria	49.6	poor clinical condition, paleness, lymphopenia	non-regenerative anemia, hyperproteinemia, azotemia
31	3	mild seborrhea/hypotrichosis, mild papules/nodules, mild lymphadenopathy, lymphopenia	49.6	arthritis, neutropenia	-
13[1]	3	mild seborrhea/hypotrichosis, mild skin ulcer, hypalbuminemia, hyperglobulinemia	22.5	proteinuria, lymphadenopathy, edema	-
13[2]	9	mild seborrhea/hypotrichosis, mild skin ulcer, lymphadenopathy, mild hypalbuminemia, hyperglobulinemia, proteinuria	37.2	edema	lymphadenopathy, proteinuria

TE, ELISA test unit (results >12 TE were considered positive); [1] first relapse during the observation period; [2] second relapse during the observation period
