# Supplementary material for: Importance of Different Parameters for Monitoring Dogs with Leishmania infantum Infections in a Non-Endemic Country
Source: Pathogens. 2025 Dec 12;14(12):1282. doi: 10.3390/pathogens14121282 (PMC12735808; doi:10.3390/pathogens14121282)
Supplement: Supplementary file 1 [file pathogens-14-01282-s001.zip › Supplementary_material.pdf]

## Supplementary materials

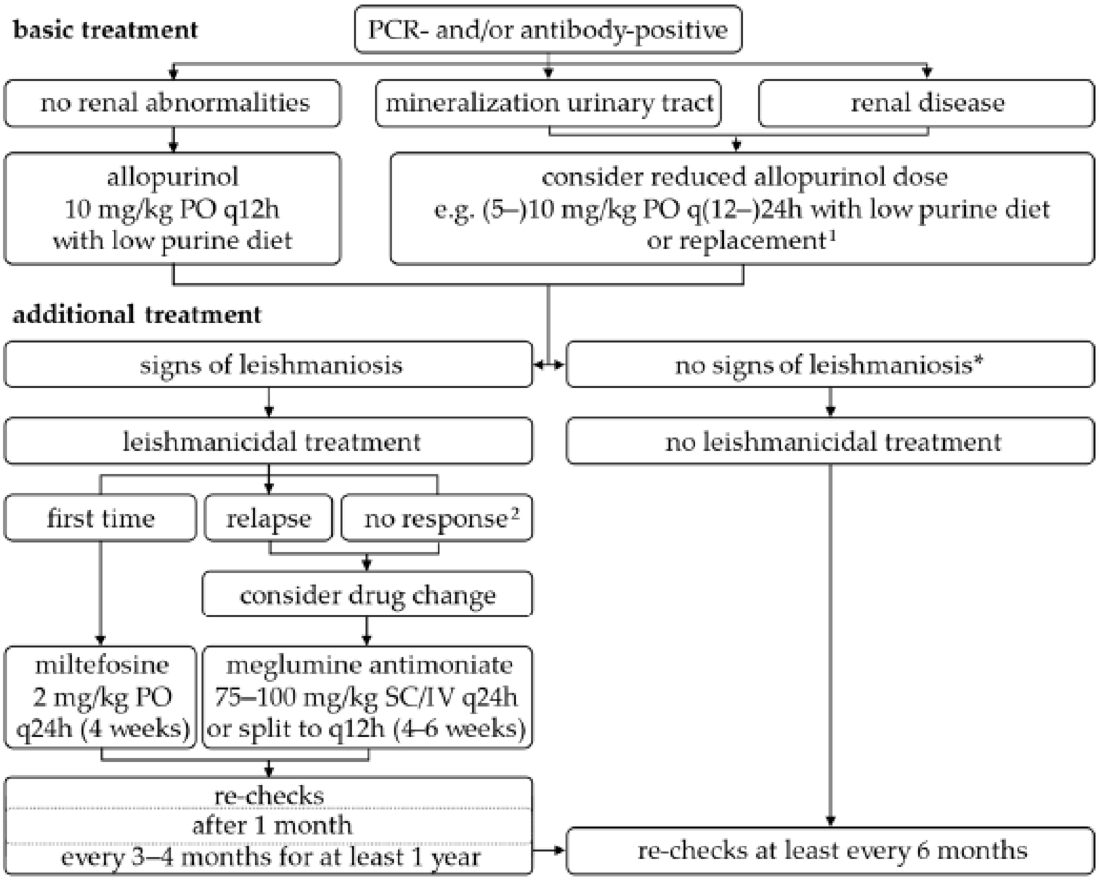


**Figure S1.** Treatment of the 52 dogs included in the study guided by the therapeutic tree for dogs with *Leishmania* infections in non-endemic areas according to Kaempfle et al., 2025 [21]. * consideration of withdrawal after a minimum duration of 6 months, in case of complete remission and markedly decreased antibody level/no antibodies; [1] e.g., with domperidone or dietary nucleotides with active hexose correlated compound; [2] no improvement or worsening of clinical and/or laboratory signs within 4 weeks after end of treatment; IV; intravenous administration; kg, kilograms; mg, milligrams; PCR, polymerase chain reaction; PO, peroral administration; q12h, every 12 h; q24h, every 24 h; SC, subcutaneous administration.

| | M0 | M3 | M6 | M9 | M12 |
|---|---|---|---|---|---|
| **dog 1** | | | | | |
| **dog 2** | R | | | | |
| **dog 3** | | | | | |
| **dog 4** | | | | | |
| **dog 5** | R | | | | |
| **dog 6** | | | | | |
| **dog 7** | | | | † | |
| **dog 8** | | | | | |
| **dog 9** | | | | | |
| **dog 10** | | | | | |
| **dog 11** | | | | | |
| **dog 12** | | | | | |
| **dog 13** | | | R | | R |
| **dog 14** | | | | | |
| **dog 15** | | | | | |
| **dog 16** | | | | | |
| **dog 17** | | | | | |
| **dog 18** | | | | | |
| **dog 19** | | | | | |
| **dog 20** | | R | | † | |
| **dog 21** | | | | | |
| **dog 22** | | | | | |
| **dog 23** | | | | | |
| **dog 24** | | | | | |
| **dog 25** | | | | | |
| **dog 26** | | | | | |

| | M0 | M3 | M6 | M9 | M12 |
|---|---|---|---|---|---|
| **dog 27** | | | | | |
| **dog 28** | | | | | |
| **dog 29** | | | | † | |
| **dog 30** | | | | | |
| **dog 31*** | | | R | R | |
| **dog 32** | | | | | |
| **dog 33** | | | | | |
| **dog 34** | | | | | |
| **dog 35** | | | | | |
| **dog 36** | | | | | |
| **dog 37** | R | | | | |
| **dog 38** | R | | | | |
| **dog 39** | R | | | | |
| **dog 40** | | | | | |
| **dog 41** | | | | | |
| **dog 42** | | | | | |
| **dog 43** | | | | | |
| **dog 44** | | | | | |
| **dog 45** | | | | | |
| **dog 46*** | R | R | R | | |
| **dog 47** | | | | | |
| **dog 48** | R | | | † | |
| **dog 49** | | | | | |
| **dog 50** | | | R | | |
| **dog 51** | | | | | |
| **dog 52** | | | | | |

**Figure S2**. Occurrence of upcoming relapse of canine leishmaniosis in 52 dogs during the one-year study period. Black box, study withdrawal; cross, death; M, month; red box, visit preceding a relapse; R, relapse; scattered box, no follow-up data; *dogs with untreated relapse
